# Janus kinase inhibitors in palmoplantar pustulosis: a mixed-methods feasibility (JAKPPPOT) trial protocol

**DOI:** 10.1136/bmjopen-2025-106361

**Published:** 2025-08-21

**Authors:** David Gleeson, Sarah Chapman, Helen McAteer, April Qin, John Gregory, Jade Pizzato, Kingsley Powell, Manpreet K Sagoo, Weiyu Ye, Ann Naylor, Lucy Moorhead, Andrew E Pink, Richard Woolf, Jonathan Barker, James B Galloway, Suzie Cro, Satveer K Mahil, C H Smith

**Affiliations:** 1 St. John's Institute of Dermatology, Guy's & St. Thomas' NHS Foundation Trust and King’s College London, London, UK; 2 Centre for Adherence Research and Education, King’s College London, London, UK; 3 Psoriasis Association, Northampton, Northamptonshire, UK; 4 Patient and Public Involvement Advisory Group, JAKPPPOT Trial, King’s College London, London, UK; 5 Centre for Rheumatic Disease, Kings College London, London, UK; 6 Clinical Trial Statistics, Imperial College London Faculty of Medicine, London, UK

**Keywords:** Psoriasis, Adult dermatology, Clinical trials, Feasibility Studies

## Abstract

**Background:**

Palmoplantar pustulosis (PPP) is a rare, debilitating inflammatory skin disease involving painful pustules on the palms and soles. Janus kinase (JAK) inhibitors target pathways relevant to PPP disease biology but also confer a risk of major adverse cardiovascular events and malignancy in certain ‘at risk’ individuals; this includes those with PPP given prevalent smoking and cardiovascular risk factors in the PPP population. The feasibility of JAK inhibitor therapy for PPP requires assessment prior to a randomised controlled trial evaluation of drug efficacy and safety for this indication.

**Methods and analysis:**

The ‘Janus kinase inhibitors in palmoplantar pustulosis: a mixed-methods feasibility’ trial is an open-label, single-centre, single-arm, mixed-methods feasibility trial of JAK inhibition in PPP (REC reference: 24/NE/0147; ISRCTN61751241). Participants (n=20) will receive 8 weeks of treatment with the JAK inhibitor upadacitinib (‘Rinvoq’, 30 mg, once daily). Qualitative semistructured interviews (up to n=40) will be undertaken with trial participants, trial decliners and healthcare professionals. The primary outcome will be a composite assessment of feasibility across three domains: recruitment, adherence and acceptability, using a mixed-methods analysis approach. Secondary objectives include the identification of trial recruitment optimisation strategies, using the ‘Quintet Recruitment Intervention’, and the generation of an indication of effect size on disease severity (measured using the Palmoplantar Pustulosis Psoriasis Area and Severity Index) to inform future sample size calculations. Historic placebo control data from the Anakinra for Pustular Psoriasis: Response in a Controlled Trial (National Institute of Health and Social Care reference: 13/50/17; Research Ethics Commitee reference: 16/LO/0436) will be used as the effect size comparator. Study recruitment will be undertaken over a 24-month period, commencing in November 2024.

**Ethics and dissemination:**

This study has been approved by the Newcastle North Tyneside 2 Research Ethics Committee, 24/NE/0132. Our findings will inform the feasibility of a future adequately powered RCT evaluating the efficacy of JAK inhibitor therapy in PPP.

**Trial registration number:**

ISRCTN61751241.

**STRENGTHS AND LIMITATIONS OF THIS STUDY**

- The Quintet Recruitment Intervention and use of historic placebo controls may generate generalisable knowledge on trial recruitment and delivery in rare disease research.
- The study aim is to evaluate feasibility; future work will be required to establish the clinical effectiveness of Janus kinase inhibitor therapy in palmoplantar pustulosis.
- This is a single-arm, single-centre study conducted in a tertiary care setting which may limit the generalisability of findings. ⁠

## Background

 Palmoplantar pustulosis (PPP) is a rare, chronic, immune-mediated skin disease, characterised by painful, intensely inflamed skin on the palms and soles, studded with sheets of pustules. The prevalence of PPP is estimated as 6.4–89.4/100 000 population, with a higher preponderance in Asia, and among females.^1^ It typically presents in middle age and is closely associated with a history of smoking.^2^

Conventional systemic and targeted biologic immunomodulatory therapies display limited efficacy in PPP, posing a significant therapeutic challenge, and there are no currently licensed treatments for PPP in the UK.^3^ Transcriptomic studies have identified strong T2-mediated and T17-mediated inflammatory signatures in the pathogenesis of PPP.^4^ The simultaneous activation of multiple immune pathways may account for the limited therapeutic efficacy of targeted biologic therapies in this condition.

Janus kinase (JAK) inhibitors are a novel class of targeted immunomodulators that are increasingly used in medical specialities, including rheumatology (eg, psoriatic arthritis), gastroenterology (eg, ulcerative colitis), and dermatology (eg, atopic eczema).^5^ JAK inhibitors exert broad immunosuppressive effects via pleiotropic inhibitory effects across several intracellular inflammatory cascades, providing a strong potential rationale for their use in PPP. Consistent with this are case reports and case series supporting their potential as a therapeutic option in PPP, with a recent critical appraisal of the literature identifying 39 cases of near/complete skin clearance following treatment with different JAK inhibitors.^6^ However, there is currently a lack of trial evidence to support this management approach in PPP.

Given the growing clinical evidence and a plausible scientific underpinning to support the use of JAK inhibitors as a treatment for PPP, further investigation in a trial setting is warranted. However, the feasibility of an adequately powered definitive randomised controlled trial (RCT) assessing the efficacy of JAK inhibitor therapy in PPP is unclear, due to several key challenges.

The first challenge relates to recent safety concerns regarding JAK inhibitor therapy following a randomised, open-label, non-inferiority, post-authorisation trial of 2911 patients with rheumatoid arthritis. This study evaluated the safety and efficacy of the JAK inhibitor tofacitinib compared with tumour necrosis factor (TNF) inhibitor therapy in patients with rheumatoid arthritis who were 50 years of age or older and had at least one additional cardiovascular risk factor.^7^ During a median follow-up of 4 years, the incidence of major adverse cardiovascular events (MACEs) was higher with combined tofacitinib (3.4%; 98 patients) than with a TNF inhibitor (2.5%; 37 patients). Non-inferiority of tofacitinib compared with TNF inhibitor therapy was not shown (HR, 1.33; 95% CI: 0.91 to 1.94), because the upper boundary of the 95% CI was more than 1.8 (the pretrial stated non-inferiority cut-off). Similarly, the incidence of cancers (excluding non-melanoma skin cancer) was higher with tofacitinib (4.2%; 122 patients) than with a TNF inhibitor (2.9%; 42 patients). Non-inferiority of tofacitinib compared with TNF inhibitor therapy was not shown (HR, 1.48; 95% CI, 1.04 to 2.09), with the upper boundary of the 95% CI more than 1.8 again.

Tofacitinib is a pan-JAK inhibitor, and it is not currently known how these safety signals relate to more selective JAK inhibitors, such as upadacitinib (a JAK-1 selective inhibitor). A recent meta-analysis found that there was no increased risk of malignancy associated with JAK inhibitor use when compared with placebo, highlighting the need for further research and long-term follow-up safety data in this area.^8^ Nonetheless, the recent safety concerns have prompted the introduction of new guidance from regulatory bodies cautioning the use of JAK inhibitors in at-risk populations (age >65 years; current or long-term former smokers; those with other cardiovascular or malignancy risk factors).^9^

These safety signals are highly relevant in the context of PPP. The prevalence estimate of smoking among the PPP population is 78.2%, and cumulative pack-year history and longer duration of smoking are associated with a greater risk of disease development.^10^ The current regulatory framework means that JAK inhibitors may be relatively contraindicated in a substantial proportion of people with PPP, and both clinicians and people with PPP may be reluctant to use JAK inhibitors in light of these potential risks. Therefore, successful participant recruitment to an RCT may thus be difficult to achieve.

This recruitment challenge is especially pertinent given PPP is a rare disease. Poor trial recruitment is a prominent issue in the study of rare diseases, leading to research failure and increased costs. Only half of publicly funded trials in the UK between 1997 and 2020 achieved their original recruitment target, and a third required an extension.^11^ Given these recruitment challenges and acceptability concerns, a feasibility assessment of the use of JAK inhibitor therapy in PPP is warranted prior to a definitive adequately powered RCT.

Poor medication adherence is also a prevalent issue in chronic diseases. It is estimated that roughly 50% of patients with a chronic disease do not take their medication as prescribed.^12^ Given the acceptability concerns highlighted, an exploration of adherence in this context is warranted, especially considering the high cost of JAK inhibitors.

An exploratory feasibility trial also provides the opportunity to explore innovative trial recruitment and efficiency strategies, such as the Quintet Recruitment Intervention (QRI).^13^ This two-phase, recruitment-focussed, quality improvement methodology has been used in at least 23 studies since 2016, ranging from surgery through to oncology, and in both feasibility and definitive trial settings. It has yet to be used in an inflammatory disease trial, or in dermatology, and PPP provides an exemplar rare disease in which to trial this approach.

Another strategy to enhance trial efficiency is the use of historic controls. Historic controls are individuals studied in a previous trial used as a comparison group in a future study that has no control group.^14^ Reusing data from the placebo arms of previous RCTs offers the potential to minimise risks, cost and inconvenience by reducing enrolment time, decreasing participant numbers, minimising potential bias due to dropout, accelerating trial completion and thus reducing time to treatment approval.^15^ Historic placebo controls have yet to be used more widely in the context of inflammatory diseases. We will pilot the use of historic controls in this setting, incorporating historic control data from the placebo group of the Anakinra for Pustular Psoriasis: Response in a Controlled Trial (APRICOT) as the comparator group. The APRICOT investigated the use of anakinra in the management of PPP against a placebo control and was conducted from 2015 to 2020.^16^ We have followed guidance from a recently published roadmap on the use of historic controls, mirroring the APRICOT processes and eligibility criteria in the proposed trial, ensuring the robustness of this approach.^17^

## Objective

The ‘Janus kinase inhibitors in palmoplantar pustulosis: a mixed-methods feasibility (JAKPPPOT)’ trial is an open-label, single-arm study which aims to determine the feasibility of JAK inhibitor therapy in PPP, via a composite, mixed-methods assessment of three domains: recruitment, adherence and acceptability. This study will provide an indication of effect size using historical control data from the APRICOT study and will identify trial optimisation strategies to bolster the recruitment in a definitive trial.^16^

## Methods

This protocol has been prepared and reported in accordance with the Standard Protocol Items: Recommendations for Interventional Trials (SPIRIT) guidance.^18^ The trial SPIRIT checklist is available (online supplemental file 1).

### Study design

**Figure 1:**
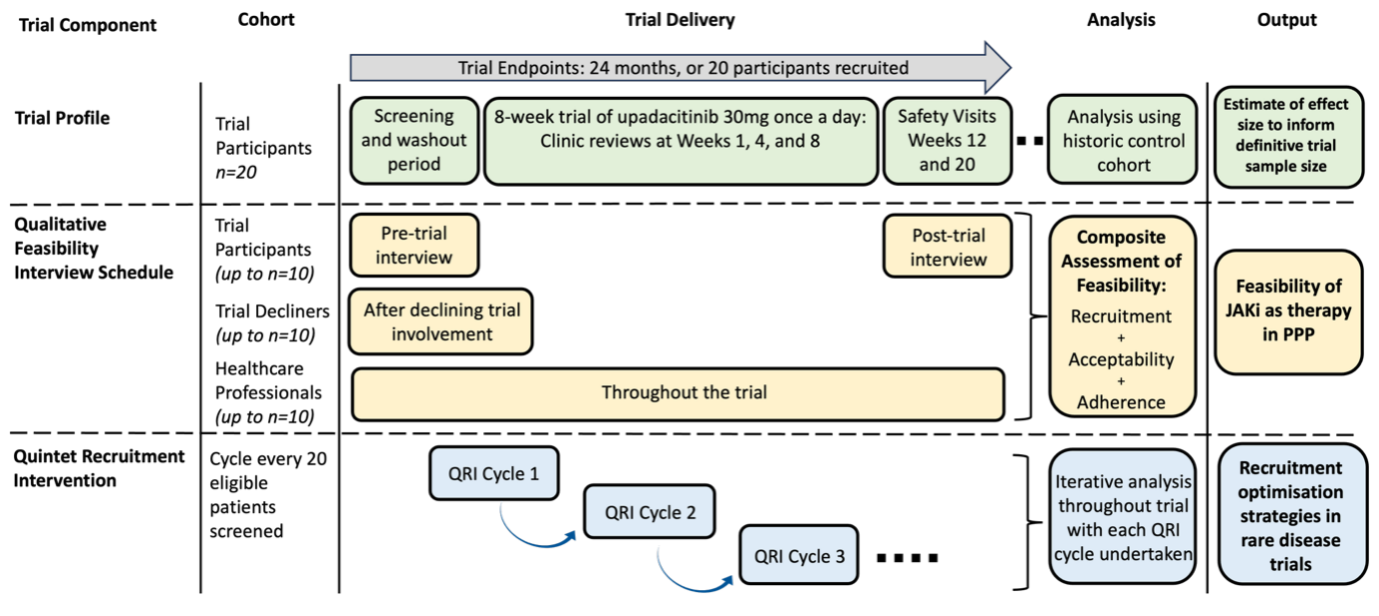
Overview of the JAKPPPOT study design. JAKi, Janus kinase inhibitor; JAKPPPOT, Janus kinase inhibitors in palmoplantar pustulosiAnus Kinase inhibitors in PalmoPlantar PustulOsis: a mixed-methods feasibility; PPP, palmoplantar pustulosis; QRI, Quintet Recruitment Intervention.

JAKPPPOT is an open-label, single-arm, mixed-methods feasibility trial, conducted at St John’s Institute of Dermatology, Guy’s and St Thomas’ NHS Foundation Trust, UK (figure 1). Participants (n=20) will receive 8 weeks of treatment with the JAK inhibitor upadacitinib (‘Rinvoq’, 30 mg, once-a-day). Integrated qualitative semistructured interviews (up to n=40) will be undertaken with trial participants, trial decliners and healthcare professionals. Recruitment optimisation strategies will be identified and implemented via the QRI process incorporated into this trial. The study endpoint is 20 participants successfully recruited, or 24 months elapsing, whichever occurs first.

### Study population

The study population is adult patients (≥18 years) with a dermatologist-confirmed diagnosis of PPP requiring systemic immunomodulatory therapy. The full inclusion and exclusion criteria are listed in table 1. In line with the Medicines and Healthcare products Regulatory Agency’s (MHRA) recent advice, upadacitinib will only be used in the following cohorts if no suitable treatment alternatives are available^9^:

**Table 1:** Inclusion and exclusion criteria

Inclusion criteria	Exclusion criteria
Adults (18 years and over) with a diagnosis of palmoplantar pustulosis* made by a trained dermatologist with disease of sufficient impact and severity to require systemic therapy Disease duration of ≥6 months, not responding to an adequate trial of topical therapy including very potent corticosteroids Evidence of active pustulation on palms and/or soles At least moderate disease based on a Palmoplantar Pustulosis Investigator Global Assessment Able to give written, informed consent to participate Willing and able to comply with scheduled visits, treatment plan, laboratory tests and other study procedures.	A history of malignancy of any organ system (other than treated, localised non-melanoma skin cancer), treated or untreated, within the past 5 years A history of provoked or unprovoked venous thromboembolism (deep vein thrombosis or pulmonary embolism), unless actively treated with long-term anticoagulation Previous systemic treatment with a JAK inhibitor A history of recurrent bacterial, fungal or viral infections which, in the opinion of the principal investigator, present a risk to the patient Evidence of active infection or untreated latent tuberculosis HIV positive Active or untreated Hepatitis B or C Use of therapies with potential or known efficacy in psoriasis during or within the specified timeframe before treatment initiation as listed in the washout section Moderate renal impairment (CrCl < 50 mL/min) Neutropenia (<1.5×10^9^/L) Thrombocytopenia (<150×10^9^/L) Moderate hepatic disease and/or raised hepatic transaminases (ALT/AST) > 2 x upper limit of normal at baseline. Patients who fail this screening criterion may still be considered following review by a hepatologist and confirmed expert opinion that study entry is clinically appropriate. Live vaccinations within 3 months prior to the start of study medication, and no planned live vaccinations during the trial and up to 3 months following last dose Women who are pregnant, breast feeding or of childbearing potential not on adequate contraception Male participants who are not willing to use highly effective methods of contraception when engaging in sexual activity with a female of childbearing potential Any condition where, in the opinion of the investigator, the investigational medical product would present risk to the patient. Unable to give written, informed consent. Unable to comply with the study visit schedule Known hypersensitivity to upadacitinib and/or its excipients (SmPC 6.1) Receipt of any of the following within the specified timeframe before treatment initiation (baseline, visit 1): Topical treatments likely to impact signs and symptoms of psoriasis (eg, potent/very potent corticosteroids, vitamin D analogues, calcineurin inhibitors) within 2 weeks Systemic immunosuppressants (eg, methotrexate, ciclosporin, acitretin) within 4 weeks Phototherapy (UVB TL01, UVB, PUVA, UVA1) within 4 weeks Etanercept or adalimumab within 4 weeks Other biologic therapies (infliximab, certolizumab, ustekinumab, secukinumab, ixekizumab, risankizumab, bimekizumab, brodalumab, tildrakizumab, guselkumab) within 3 months Other investigational drugs within 4 months or five half-lives (whichever is longer) Other immunosuppressant/immunomodulatory therapies including intra-articular steroids within 30 days or five half lives (whichever is longer)

* Different forms of psoriasis can co-present together (eg, chronic plaque psoriasis, acrodermatitis of Hallopeau, generalised pustular psoriasis). A concomitant diagnosis of a different type of psoriasis will not be a contraindication to eligibility.

ALT, alanine aminotransferase; AST, aspartate aminotransferase; CrCl, creatinine clearance; SmPC, summary of product characteristics.

1. Age 65 years or older.
2. Patients with a history of atherosclerotic cardiovascular disease or other cardiovascular risk factors (such as past long-term smokers).
3. Patients with malignancy risk factors (eg, current malignancy or history of malignancy).

Any decision to treat patients in these cohorts will be made following discussion with the chief investigator and will be based on individualised patient factors.

### Intervention

Participants will receive 8 weeks of treatment with upadacitinib (‘Rinvoq’, 30 mg, once daily, oral tablet). In certain patient cohorts, outlined in Section 4.4 of the summary of product characteristics, the investigational medical product (IMP) dose may be moderated to 15 mg, once daily, oral tablet. These cohorts include patients at higher risk of venous thromboembolic events, MACEs and malignancy. Any decision to reduce the dose will be made following a discussion with the chief investigator.

Upadacitinib has been selected as the exemplar JAK inhibitor of choice for this trial. Upadacitinib is a JAK1 inhibitor, with some JAK2 inhibition, which shows good efficacy in the management of atopic eczema (an IL-4/T2-mediated disease).^19^ Its inhibitory action across both JAK1- and JAK2-mediated pathways provides a strong mechanistic rationale for its use in PPP.^4^ To date, there have been over 50 reported cases indicating the efficacy of upadacitinib in PPP, contributing to a growing clinical interest in its use.^20 21^ The findings of this study will inform the decision regarding drug choice for a future definitive RCT.

### Comparator

Retrospective placebo-controlled data from the APRICOT (National Institute of Health and Social Care Research [NIHR]reference: 13/50/17; Research Ethics Committee [REC] number: 16/LO/0436; International Standard Registered Clinical/soCial sTudy Number [ISRCTN] number: ISCRTN13127147) will be used to generate an indication of effect size for the purpose of estimating the required sample size of a definitive RCT.^16^ Placebo controls in the APRICOT previously received 0.67 mL of vehicle solution daily through self-administered subcutaneous injection over 8 weeks. The IMP in APRICOT was anakinra (Kineret; SOBI, Stockholm, Sweden; 100 mg/0.67 mL daily delivered via self-administered subcutaneous injection).

### Outcomes

#### Primary outcomes

The following outcomes will be assessed after 24 months of recruitment, or when the recruitment target of 20 participants is achieved (whichever occurs first):

1. Overall proportion of potential participants identified who enrolled in the trial, termed the ‘recruitment rate’.
  - ‘Potential participants identified’ is defined as those who have direct contact with the research team.
2. Overall proportion of participants achieving ‘good adherence’ (proportion of days covered >80%) during the trial.
  - ‘Proportion of days covered’ is defined as the total medication taken during the 8-week trial period as a proportion of the total medication dispensed.
3. Overall proportion of participants who view JAK inhibitor therapy in PPP to be ‘acceptable’.
  - Acceptability will be measured based on responses to the following question at the end of the 8-week treatment period: ‘Overall, how acceptable was taking upadacitinib to you?’
  - A 5-point Likert scale will be used, with answers dichotomised into acceptable (acceptable/completely acceptable) or unacceptable (completely unacceptable/unacceptable/neutral)

#### Secondary outcomes

The following outcomes will be assessed after 24 months of recruitment, or when the recruitment target of 20 participants is achieved (whichever occurs first):

1. Total number of potential identified participants who have contact with the research team.
2. Speed of participant identification (participants identified/month).
3. Speed of participant recruitment (participants identified/month).
4. Total number of ineligible participants.
5. Reasons for ineligibility.
6. Recruitment of 20 participants within 24 months.
7. Overall mean change in Palmoplantar Pustulosis-Psoriasis Area and Severity Index (PP-PASI) over the 8-week treatment period, adjusted for baseline PP-PASI.^22^
8. Placebo control data from the APRICOT (2015–2020, n=31 with complete data) will be used as a comparator.

The following outcome is assessed at the end of each phase of the QRI approach (new phase for every 20 eligible patient participants approached throughout the trial):

- Change in recruitment rate with each phase of the QRI approach.

#### Exploratory outcomes

The following outcomes are assessed after 24 months of recruitment, or when the recruitment target of 20 participants is achieved (whichever occurs first):

1. Evaluation of trial recruitment diversity and inclusion.
  - Participant demographics will include age, sex, ethnicity and socioeconomic background.
2. Accuracy of disease severity assessments of photographs compared to in-person assessments.
  - PP-PASI and Palmoplantar Pustulosis-Investigator Global Assessment (PPP-IGA) grading of photographs will be compared with in-person disease severity assessments.
3. Overall mean change in total pustule count over the 8-week treatment period, compared to baseline adjusted for baseline total pustule count.
  - Placebo control data from the APRICOT (2015–2020, n=31 with complete data) will be used as a comparator.
4. Overall mean change in fresh pustule count over the 8-week treatment period, compared to baseline adjusted for baseline fresh pustule count.
  - Placebo control data from the APRICOT (2015–2020, n=31 with complete data) will be used as a comparator.
5. Overall proportion of participants achieving clearance of PPP over the 8-week treatment period.
  - Clearance will be defined as a PPP-IGA of 0/1.
  - Placebo control data from the APRICOT (2015–2020, n=31 with complete data) will be used as a comparator.
6. Overall mean change in chronic plaque psoriasis severity (if applicable) over the 8-week treatment period, adjusted for baseline chronic plaque psoriasis severity.
  - Severity will be assessed using the PASI.^23^
  - Placebo control data from the APRICOT (2015–2020, n=16 with complete data) will be used as a comparator.
7. Overall mean change in participants’ symptoms and quality of life over the 8-week treatment period, adjusted for baseline. Dermatology Life Quality Index, Patient Global Assessment and European Quality of Life 5 Dimensions 5 Level Version (EQ5D-5L) will be used to assess participants’ symptoms and quality of life
  - Placebo control data from the APRICOT (2015–2020, n=31 with complete data) will be used as a comparator.
8. Overall proportion of participants achieving a 50% reduction in PP-PASI score (PPPASI50) over the 8-week treatment period, compared to baseline (visit 1).
  - Placebo control data from the APRICOT (2015–2020, n=31 with complete data) will be used as a comparator.

### Adverse events monitoring

Adverse events will be tabulated and described for all subjects who received at least one dose of study medication. Adverse events will be summarised by event type, including information on the number with at least one event and the number of events to account for recurrent events.

### Sample size rationale

We will aim to recruit 20 participants, in keeping with other published feasibility studies.^24 25^ Over a 24-month recruitment period, we anticipate approaching a total of around 100 potentially eligible individuals. Based on previous experience, we anticipate that the actual recruitment rate would be no less than 20%, so this would provide us with at least 20 participants.

With 100 approached participants, if 20 (20%) are recruited into the trial, this would allow us to estimate the 95% CI for the recruitment rate with precision of at least ±8%. Assuming 75% of participants adhere to treatment at least 80% of the time (as observed in APRICOT), a sample size of 20 participants would allow us to estimate ≥80% treatment adherence with a 95% CI: 51% to 91%. With 20 participants on treatment, JAKPPPOT will estimate a PPPASI50 in the JAK inhibitor arm with precision of at least ±3.43 PP-PASI points. This is based on figures derived from the APRICOT (mean baseline PP-PASI: 17.8; SD for the PP-PASI change at 8 weeks in active arm: 7.32).^16^ A PPPASI50 is an outcome that has been analysed as a primary/secondary endpoint in several PPP trials and was described as ‘treatment success’ during patient and public involvement (PPI) discussions with people with PPP.^26^ The PP-PASI reduction in the treatment arm will inform the sample size calculation for a larger definitive trial.

### Progression criteria and rationale

Progression criteria have been outlined to determine whether it is appropriate to progress to a subsequent adequately powered RCT, shown in table 2. Findings from the primary and secondary quantitative outcomes will be incorporated with the qualitative findings to inform decisions regarding an ‘intermediate’ results in any domain (ie, upgrade to feasible or downgrade to non-feasible). All recommendations will be reviewed by the trial steering committee (TSC). For criteria judged to be intermediate or non-feasible, the study team will examine the likely causes and consider whether a mitigation strategy could be successfully implemented.

**Table 2:** Progression criteria for the JAKPPPOT trial

Domain	Non-feasible	Intermediate	Feasible
Recruitment: proportion of potential participants identified who enrolled in the trial	<20%	20–40%	>40%
Adherence: proportion of participants achieving ‘Good’ adherence (>80% of medication taken as prescribed)	<20%	20–80%	>80%
Acceptability: proportion of participants who view JAK inhibitor therapy in PPP to be ‘acceptable’	<20%	20–80%	>80%

JAK, Janus kinase; JAKPPPOT, Janus kinase inhibitors in palmoplantar pustulosis: a mixed-methods feasibility; PPP, palmoplantar pustulosis.

### Recruitment

A low recruitment rate is assumed, in line with the APRICOT study (17% overall recruitment rate).^16^ Factors in favour of a higher recruitment rate than the APRICOT are the single-arm trial design and the use of an oral tablet medication versus a daily subcutaneous injection with a high burden of injection site reactions. Factors negatively impacting recruitment are that this is a feasibility study rather than an efficacy study, there is no associated open-label extension and the potential side effect profile of upadacitinib. Optimisation strategies will be implemented through the trial to improve recruitment.

Secondary quantitative outcomes will be incorporated into the feasibility assessment, for example, failure to recruit 20 participants within 24 months would be an indicator of non-feasibility. If recruitment cannot be achieved in the context of an open-label trial, it is strong evidence against the feasibility of an adequately powered RCT.

### Adherence

Adherence will be assessed by counting any remaining medication via inspection of medication packaging at the end of each 4-week period (visit 3 and visit 4) to determine the proportion of days covered (total medication taken/total medication dispensed). If the packaging is unavailable, participants’ responses to the Voils DOSE-Nonadherence measure will be used as a surrogate.^27^

A high adherence rate is expected for several reasons. The tablet is easy to administer, and there is a relatively high burden of clinic visits over the 8-week treatment period. This will remind participants to take the medication and allow any compliance issues to be identified and addressed quickly.

Previous studies have demonstrated an association between an adherence rate of >80% and improved outcomes, when compared with an adherence rate of <80%, in certain disease contexts (eg, following a myocardial infarction).^28 29^ An 80% adherence threshold is often used, therefore, as a key measure of adherence in the context of clinical trials.^30^

The optimal threshold for adherence will differ depending on the specific disease, medication and population in question. Given the paucity of current literature regarding the use of upadacitinib in the management of PPP, it is not currently possible to determine the necessary adherence threshold for this specific context, and so the accepted threshold of >80% adherence rate has been used.^30^ Given the small numbers of trial participants, a wide intermediate progression criteria window has been specified, allowing findings from the integrated qualitative study to be incorporated into the analysis.

### Acceptability

The recent safety concerns regarding the use of this drug class among those at risk of cardiovascular disease may render them unacceptable to people with PPP, a population in which smoking is prevalent.^7 31^ However, there are several factors that may mitigate these concerns. There is a growing body of evidence to support the safety and efficacy of JAK inhibitors in the management of other medical conditions, with upadacitinib well-tolerated in the context of atopic dermatitis.^32^ The ease of administration is also a factor cited by patients in support of their acceptability.^33^ Given the lack of effective treatment options available for this condition, resulting in marked unmet clinical need, participants may be more willing to consider treatment with a JAK inhibitor in spite of side effect concerns.^3^ Finally, MHRA guidance has been incorporated into the study design, ensuring those most at risk from JAK inhibitor treatment are not eligible to take part. Given the small number of trial participants, a wide intermediate progression criteria window has been specified, allowing findings from the integrated qualitative study to be incorporated into the analysis.

### Participant recruitment

Potentially eligible trial participants will be identified via a number of means, including routine care visits, existing participants of related research studies who have consented to recall and self-referral following advertisement through national (eg, Psoriasis Association) and regional communication channels (newsletters, websites and social media). Participants can choose to participate in the trial and/or the semistructured interviews. Written, informed consent will be obtained by a physician (online supplemental file 2).

### Study procedures

The full study activities at each visit are outlined in table 3. The patient participant pathway consists of three periods: a screening period, a treatment period and a follow-up period. The screening period is up to a maximum of 3 months. Patients who fail the screening process may be re-screened if clinically appropriate.

**Table 3:** Schedule of trial activities

	Screening (<3 months between screening and baseline)	Treatment period				Follow-up	Safety follow-up
**Allowed visit window:** **+** **3 days**	**Visit 0**	**Visit 1**	**Visit 2**	**Visit 3**	**Visit 4**	**Visit 5**	**Visit 6**
		Baseline	(week 1)	(week 4)	(week 8)	(week 12)	(week 20)
	Study enrolment	Treatment initiation			Treatment end	Study end	
Written informed consent	X						
Inclusion/exclusion eligibility check and sign-off	X	X					
Demographics	X						
Medical history	X	X					
Social history	X						
Family history	X						
PPP history	X						
Clinical phenotyping of disease/concomitant psoriasis	X						
Physical examination	X						
Vital signs*	X	X	X	X	X	X	
PPPASI† (×2: blinded and unblinded assessment)	X	X	X	X	X	X	
Fresh pustule count†	X	X	X	X	X	X	
Total pustule count†	X	X	X	X	X	X	
PPP-IGA† (×2: blinded and unblinded assessment)	X	X	X	X	X	X	
PASI (plaque psoriasis only)	X	X	X	X	X	X	
BSA	X	X	X	X	X	X	
Patient global assessment	X	X	X	X	X	X	
DLQI		X			X	X	
EQ5D-5L		X			X	X	
DOSE-Nonadherence questionnaire			X	X	X		
Photography (palms and soles)		X	X	X	X	X	
CXR‡	X						
T-Spot.TB§	X						
HIV, HBV and HCV	X						
Safety bloods¶**	X^††^	X^††^	X	X	X	X	
bHCG (blood)‡‡	X	X			X	X	
Prescribing and dispensing trial IMP		X		X			
Retrieval of IMP packaging (pill count)				X	X		
Acceptability Questionnaire§§		X				X	
RCT scoping question						X	
Concomitant meds	X	X	X	X	X	X	X
Adverse events monitoring		X	X	X	X	X	X
**Integrated qualitative study**							
Trial participant interview	X				X		
Trial decliner interview	X						
Healthcare professional interview	Interviews will take place with healthcare professionals throughout the duration of the trial (maximum one interview per healthcare professional)						

* Vital signs include heart rate, blood pressure, oxygen saturations, respiratory rate and temperature.

† Assessed by independent blinded assessor. PPPASI and PPP-IGA were also assessed by a second assessor.

‡ CXR not indicated if participant has had a CXR in the previous 12 months for clinical purposes.

§ T-Spot.TB not indicated for those participants known to have been successfully treated for TB (completed the prescribed treatment courses) as screening test is not clinically indicated. If unsure, please seek specialist advice.

¶ Safety bloods comprise WBC, Hb, platelet count, creatinine, urea, sodium, potassium, bilirubin, aspartate aminotransferase, alanine transaminase, HbA1c and cholesterol.

** CRP to be collected at baseline (visit 1) only.

†† If the time between screening and baseline safety assessment bloods is >4 weeks (ie, for participants washing out for 3 months from biologic therapy) the participant should be asked to attend for additional safety assessment blood tests. If feasible, this should be on the same day as the baseline visit allowing for time to clinically review the results before the first treatment dose (in which case only one set of baseline safety assessment bloods should be taken); however, if not convenient, it should be scheduled within 4 weeks of the baseline visit (these may be taken by their GP). If the participant attends an extra visit for these tests, then they should also go on to complete the full baseline visit, that is, repeat the baseline safety assessment bloods as scheduled.

‡‡ bHCG not indicated or applicable for postmenopausal women.

§§ Acceptability questionnaires will be completed during Visits 1 and 5 for participants not taking part in the integrated qualitative study (qualitative study participants will complete the questionnaires during the interviews).

bHCG, beta-human chorionic gonadotropin; BSA, body surface area; CXR, chest X ray; DLQI, Dermatology Life Quality Index; EQ5D-5L, European Quality of Life 5 Dimensions 5 Level Version; Hb, haemoglobin; HBV, Hepatitis B virus; HCV, Hepatitis C virus; IMP, investigational medical product; PPP, palmoplantar pustulosis; PP-PASI, Palmoplantar Pustulosis Psoriasis Area and Severity Index; PPP-IGA, Palmoplantar Pustulosis-Investigator Global Assessment; RCT, randomised controlled trial; TB, tuberculosis.

The treatment period (visits 1–4) is 8 weeks. At the start of the treatment period, eligible patient participants will receive upadacitinib, to be administered daily as an oral tablet for 8 weeks. Participants will receive 4 weeks of medication at Visit 1 (baseline) and Visit 3 (week 4).

The follow-up period (visit 5) at week 12 will be used to assess disease relapse off study treatment, follow-up any adverse events and plan for post-trial management of their condition. Participants will also be asked the following question to scope out their views on a future RCT: ‘Would you be interested in participating in a trial similar to this with either a dummy arm or active comparison drug arm in the future?’. A final safety review (visit 6) will be carried out at week 20 to assess for any further adverse events.

### Concomitant medication

Emollient therapy and mild-moderate topical corticosteroids will be permitted throughout the trial. Investigator-directed use of potent/super potent topical corticosteroids as ‘rescue’ therapy can be used if necessary, with the volume used recorded at study visits to evaluate any potential confounding effect.

Systemic therapies that are likely to have efficacy in PPP or to compound the immunosuppressive effects of upadacitinib are prohibited. If treatment with these is essential, the participant must notify the study team and they will be withdrawn from the trial.

Guidance from the clinical trials facilitation and co-ordination group will be followed regarding effective forms of contraception during the trial.

### Data collection and management

All outcome assessments of PPP will be assessed by an independent assessor blinded to the patient participant’s position on the trial pathway (ie, screening/receiving treatment/follow-up visit) to minimise bias. They will not be involved in other aspects of the trial (eg, recruitment, qualitative interviews). There should be no need for unblinding of assessors as safety concerns will be addressed by the unblinded research team members.

A visit window of 3 days either side of the scheduled visit will be allowed. In the event that a visit falls outside the target visit window of +3 days, the visit should be scheduled as soon as possible, and the data should be entered into the intended visit. All visits will be carried out in person. In exceptional circumstances where a patient is unable to attend a study visit, a virtual visit will be carried out.

Data will be collected using a paper case report form and stored securely in an electronic data management system (CAPTURE). Personal information stored within CAPTURE that could identify individuals will remain strictly confidential. Access to the information will be restricted at all times to researchers with clear purpose or need to access it. All data held within CAPTURE is processed in accordance with the Data Protection Act 2018 and UK General Data Protection Regulation (GDPR).

Should a participant decide to withdraw from the trial, all efforts will be made to report the reason for withdrawal as thoroughly as possible and they will be encouraged to continue to provide outcome data. All data collected to date will be retained.

We will seek participant consent in the informed consent form for their de-identified study data to be shared with research collaborators running other research studies in our organisation and in other organisations.

### Statistical analysis

Baseline characteristics will be summarised by using suitable measures of central tendencies. For continuous data, means/medians and SD/IQR will be reported depending on the data distribution, and frequencies with proportions will be reported for categorical data. The primary feasibility outcomes will be reported as proportions with a 95% CI. No formal hypothesis testing will be conducted as this is a feasibility study. A detailed statistical analysis plan has been written for the analysis approach.

The mean change in PP-PASI at week 8 for the treatment arm will be estimated with a 95% CI. This analysis will follow an intention-to-treat principle and include all participants with a non-missing outcome, regardless of how much treatment was received. Every effort will be made to reduce missing data. No imputation will be made for missing data for this feasibility study. Patterns of missingness will be explored and its relationship to the outcome described.

For the comparison with the historical control data, the analysis model will be a linear (Gaussian) mixed effect model, using PP-PASI scores from weeks 1/4/8, adjusted for baseline PP-PASI. This model will include a random effect for participant and fixed effects for time, time-by-treatment group interaction and baseline PP-PASI.

Statistical analysis will be undertaken according to the latest guidelines on the use of historical controls.^16^ The trial protocol is matched to the APRICOT where possible (inclusion/exclusion criteria, study methods, measurements).^16^ The access to contemporaneous, patient-level data will enable adjustment for patients’ covariates and the involvement of a blinded assessor will mitigate the inherent risk of bias associated with an open label, single-arm design. Sensitivity analyses exploring the use of inverse probability of treatment weighting to adjust for any observed differences between the treatment cohort and the historic controls will be conducted if unbalance is observed between the treatment cohort and the historic controls to ensure the robustness of results.^34^ Given the change to the APRICOT exclusion criteria to address the recent MHRA guidance (ie, exclusion of patients over the age of 65 unless there are no suitable alternatives), a sensitivity analysis based on the subset of patients across APRICOT and JAKPPPOT who would have been eligible for both trials will be conducted if required.^9^ If previous placebo APRICOT participants are recruited into the JAKPPPOT study, an additional sensitivity analysis will be conducted, excluding their data from the APRICOT placebo cohort.

### Integrated qualitative study

Qualitative research will be integrated into the trial to provide fundamental insights into the feasibility and design of a definitive trial. Qualitative interviews will be undertaken in the following three cohorts:

1. Trial participants: semistructured interviews before and after the 8-week treatment period (up to 20 interviews).
2. Trial decliners: one semistructured interview per trial decliner (up to 10 interviews). Trial decliners are individuals who have been invited to participate in the clinical trial and then declined.
3. Healthcare professionals: one semistructured interview per healthcare professional (up to 10 interviews).

Qualitative interviews will be undertaken by an appropriate, delegated member of the research team. These interviews will be conducted either in person or online via Microsoft Teams, depending on participant preference. Interview topic guides will be used to ensure similar areas are covered with each participant, but will be sufficiently flexible to encourage the interviewees to express their own views about the study. We will aim to interview up to 10 eligible participants in each cohort, or up until data saturation has been reached (no new themes were generated after three sequential participant interviews).

The semistructured interviews will be recorded, transcribed verbatim and thematically analysed.^35^ Transcripts will be reviewed and coded thematically using the NVivo 15 software package, identifying key insights, experiences or opinions. Coded interviews will be reviewed and compared so as to characterise recurrent and/or prioritised ideas about the study and interventions. The perspectives of different participants will be compared and synthesised in this process in order to reflect on similar and different experiences. Analytic codes and themes will be tested and refined as new data are considered. The results will be triangulated with the other study findings to inform recommendations for implementing trial findings and the design of the future definitive RCT. Differences in prospective and retrospective acceptability will be explored via comparison between the pretrial and post-trial interview responses.^36^

### Quintet Recruitment Intervention

The aim of the QRI is to understand the recruitment process and how it operates in the context of the trial.^13^ Sources of recruitment difficulties can then be identified and suggestions made to change aspects of the design, conduct, organisation or training that could then lead on to improvements in recruitment.

**Figure 2:**
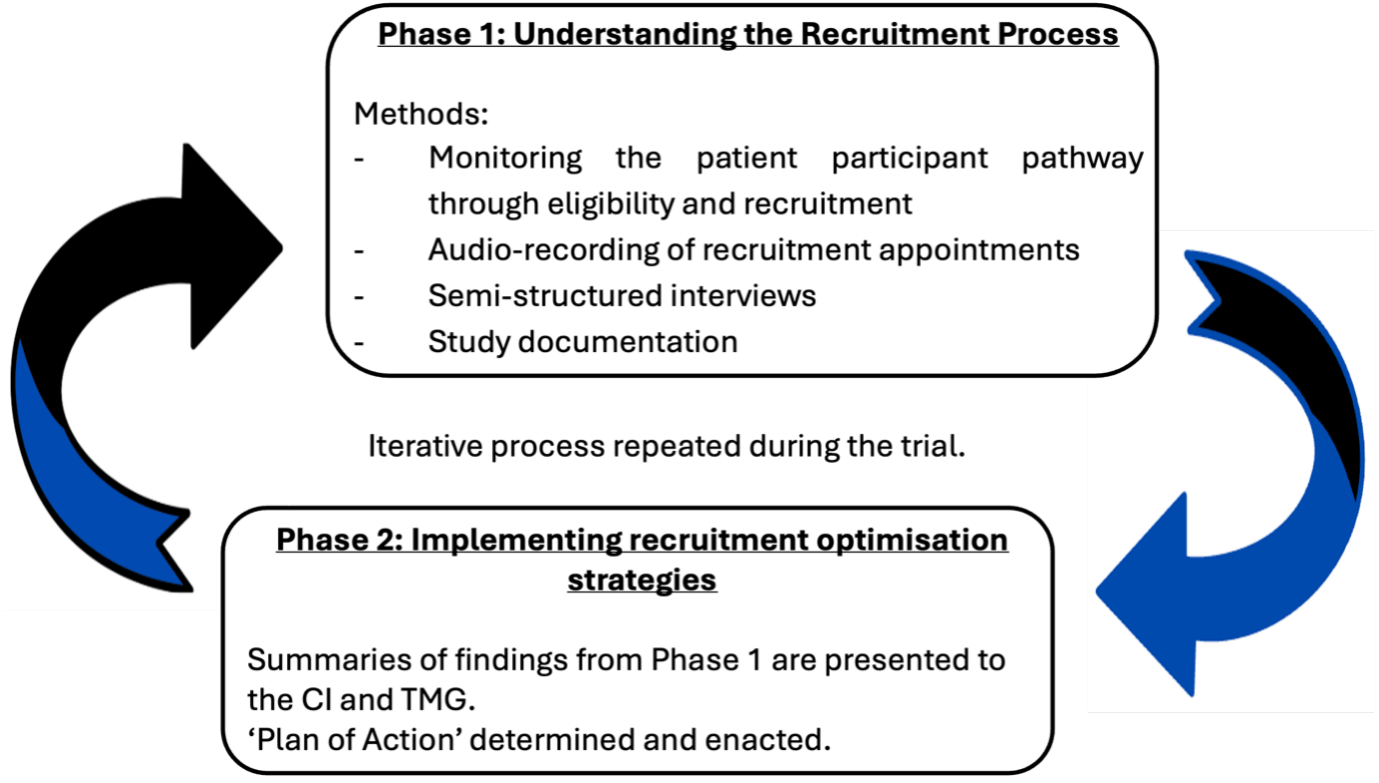
An outline of the JAKPPPOT Quintet Recruitment Intervention phases. CI, chief investigator; JAKPPPOT, Janus kinase inhibitors in palmoplantar pustulosiAnus Kinase inhibitors in PalmoPlantar PustulOsis: a mixed-methods feasibility; TMG, trial management group.

The aim of Phase 1 is to understand the recruitment process as it occurs. There are several distinct sources of information that can be used to identify recruitment difficulties about recruitment, as outlined in figure 2.

Findings of the qualitative analysis from Phase 1 will be presented to the chief investigator and trial management group (TMG). During Phase 2, a plan of action will then be drawn up and implemented to optimise recruitment.

Previous trial adaptations identified and enacted using the QRI approach include, but are not limited to^37^,^41^:

1. Discussing the interpretation of eligibility criteria and equipoise with trial site clinicians.
2. Providing QRI training and support for recruiters about how to manage patient preferences.
3. Providing suggestions to change patient information leaflets and trial literature.
4. Discussing the definition of usual care with the trial team.
5. Providing training and support for recruiters about trial design and how to approach all eligible patients.

In addition to these, possible alterations to the JAKPPPOT trial protocol that have been identified by the study team include:

1. Adjustment of the inclusion criteria to allow co-prescription of other conventional systemic therapies, such as methotrexate and ciclosporin, alongside upadacitinib.
2. Formal involvement of a wider clinical trials network to bolster recruitment efforts.
3. Production of a promotional video for distribution on social media platforms (eg, via the Psoriasis Association).
4. Incorporation of adherence support methods, such as a trial medication diary.

### Equality, diversity and inclusion

PPP has a strong female sex preponderance, and so it is likely that recruitment will mirror this gender imbalance (APRICOT consisted of 84% female participants). There will be no restrictions imposed on involvement in this study based on sex, disability, marital status, sexuality, ethnicity, religion or socioeconomic status. Travel costs will be covered to minimise the risk of cost being a barrier to participation.

The semistructured interviews will be carried out either in person or online, depending on participant preference. This has been done to ensure that potential cohorts are not excluded (eg, those with certain work commitments, language barriers and IT issues), bolstering inclusivity.

We will use our collaboration with the Psoriasis Association to ensure widespread publicity for this research, to ensure a representative demographic of participants is included. One of the exploratory aims of the study will be to carry out a retrospective evaluation of the study population to ensure trial recruitment diversity and inclusion can be optimised in advance of a future RCT.

### Patient and public involvement

This trial has been designed to address the James Lind Alliance priorities for psoriasis research, including how to induce remission, treat flares and regain control.^42^ The trial design was informed by a PPI meeting with four patients with lived experience of PPP (three women, one man), and further discussions with one patient with PPP with lived experience of treatment with a JAK inhibitor. A PPI advisory panel has been established, which will meet 6-monthly throughout the trial. Participant communication materials have been co-developed with this PPI panel. Proposed changes identified as part of the QRI process will be reviewed with PPI panel members prior to implementation. The PPI panel will review emerging findings from the trial and feedback to the TMG.

We will advertise the trial via the Psoriasis Association, the UK’s leading member organisation for people affected by psoriasis, and invite testimonials for social media, purposefully inviting underserved groups.

### Ethics approval, monitoring and dissemination

This study has been approved by the Newcastle North Tyneside 2 Research Ethics Committee (REC, reference 24/NE/0132) and the Health Research Authority (HRA). Any subsequent protocol amendments will be submitted to the REC and HRA for approval as appropriate.

Monitoring of the trial will be conducted by an independent TSC, in accordance with the MRC guidelines for Management of Global Health Trials, 2022.^43^ Given this is a small-scale, feasibility non-CTIMP (Clinical Trial of an Investigational Medicinal Product) trial using a well-established drug (upadacitinib) that has been licensed for use in the UK since 2020, the role of a data monitoring committee will be subsumed in the role of the TSC. The TSC will be comprised of an independent chair, an independent statistician, a patient representative and an independent clinical expert. No formal interim analysis with prespecified stopping guidelines will be conducted.

Results will be made available to healthcare professionals, patients, the funders and other researchers through scientific publications in peer-reviewed journals. We will also produce lay summaries, newsletters and infographics for distribution via the Psoriasis Association. Our PPI panel will be invited to contribute to study publications as co-authors.

The protocol and statistical analysis plan can be obtained by contacting the corresponding author. The study team will retain the exclusive use of data until publication of major outputs has been completed. Guy’s and St Thomas’ NHS Foundation Trust and King’s College London are co-sponsors of this research project and have shared data controller responsibilities.

## Discussion

This trial aims to provide a comprehensive mixed-methods assessment of the feasibility of JAK inhibitor therapy in PPP. Progression criteria (‘feasible’/’intermediate’/’non-feasible’) have been outlined to determine whether it is appropriate to progress to a definitive trial. Qualitative findings will be used to inform ‘intermediate’ results in any domain.

This trial employs several innovative approaches. The first is the use of placebo data from the APRICOT as historic controls. This trial will generate an indication of effect size, informing subsequent power calculations for future adequately powered clinical trials. Alongside efficiency savings, this design also mitigates the ethical concerns of giving a placebo treatment to trial participants, given the known marked burden of disease associated with PPP. The use of historic controls is established in oncology and certain rare disease trial contexts. There is one reported study in dermatology using historic controls in a comparison of surgical techniques, and none investigating drug therapies.^44^ This is, therefore, an exciting opportunity to pilot this trial design in an inflammatory dermatological setting, while also maximising the efficiency of this feasibility study.

The QRI approach represents a further opportunity for innovation. Embedding this iterative methodology within the feasibility trial will allow for an agile, adaptive approach, with the trial design and participant recruitment strategies finessed over the course of the trial. These strategies can then be incorporated into future research efforts to aid the efficiency and success of a definitive trial.

A limitation of this study is the planned single-arm, open-label trial design. Participants may be more willing to participate in a single-arm study than a RCT. To mitigate this, this topic will be explored in the integrated qualitative study, with participants asked whether they would consider participating in a definitive trial with a placebo arm in the future. Further limitations are the short duration (8 weeks) of the trial, and the lack of a planned open-label extension. These limitations were explored as part of the PPI panel discussion during the planning and design of the study and were felt to be acceptable provided they were adequately explained to potential participants prior to enrolment. This is also a single-centre study, based in a tertiary dermatology centre, and so the findings may not be generalisable to a multi-centre RCT involving secondary care centres. Barriers to implementation in other care settings will be explored in the qualitative interviews with healthcare professionals.

## Supplementary material

10.1136/bmjopen-2025-106361 online supplemental file 1

10.1136/bmjopen-2025-106361 online supplemental file 2
