# Supplementary material for: Janus kinase inhibitors in palmoplantar pustulosis: a mixed-methods feasibility (JAKPPPOT) trial protocol
Source: BMJ Open. 2025 Aug 21;15(8):e106361. doi: 10.1136/bmjopen-2025-106361 (PMC12374624; doi:10.1136/bmjopen-2025-106361)
Supplement: online supplemental file 1 [file bmjopen-15-8-s001.docx]

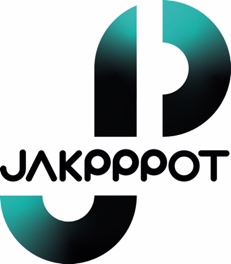


**TREATMENT CONSENT FORM**

**STUDY TITLE:** **JAKPPPOT trial**

IRAS Number: 346783

Ethics Ref: 24/NE/0147

Chief Investigator: Prof Catherine Smith

| **Site Name:** |  | **Site Number:** |  |
| --- | --- | --- | --- |
| **Principal Investigator:** |  | | |
| **Participant ID:** |  | | |

|  | **Please Initial** |
| --- | --- |
| 1. I confirm that I have read and understand the information sheet (version X.X, XX/XX/XXXX) for the above study and have had the opportunity to think about the information, ask questions and have had helpful answers that I am happy with. |  |
| 1. I understand that my participation is voluntary and that I am free to withdraw at any time, without giving a reason, without my medical care or legal rights being affected. I understand that data obtained prior to withdrawal will be kept by the research team. |  |
| 1. I give permission for the research team to access my medical records for the purposes of this research study. |  |
| 1. I understand that relevant sections of my medical notes and data collected during the study, may be looked at by individuals from the Sponsor (Guy’s and St Thomas’ NHS Foundation Trust and/or King’s College London), from regulatory authorities or from the NHS Trust, where it is relevant to my taking part in this research. I give permission for these people to have access to my data and medical records. |  |
| 1. I give permission for my personal information (including name, address, date of birth, telephone number and consent form to be passed to King’s College London so that I can take part in of the study. |  |
| 1. I agree to my General Practitioner being told that I am taking part in the study. |  |
| 1. I agree to donate blood samples and to complete trial specific activities as described in the patient information leaflet. I understand that these blood tests will be used for safety monitoring and will not be stored long-term for research use after this is complete. |  |
| 1. I give my consent for my personal data to be collected for the purposes of this research study as described in the accompanying Participant Information Leaflet. |  |
| 1. I agree to personal information from which I can be identified being held by the research team in a secure ethically approved research database. I understand that access to this personal information, from which I can be identified, will be restricted to the research team at Guy’s & Thomas’ NHS Trust. |  |
| 1. I understand that my data may be linked to relevant datasets held by national providers of healthcare data for long term follow up and health economic research, and to other research studies that I may have contributed to (e.g. the BSTOP study). |  |
| 1. I understand that my de-identified data may be used for future studies that are ethically approved. |  |
| 1. I understand that my de-identified study data (including clinical information) may be shared with other research collaborators, which may involve data being transferred outside the UK (where data laws are different) and to pharmaceutical industry partners and/or other vendors for the purposes of research for this and future ethically approved studies. |  |
| 1. I understand that data generated during this study may be used for future commercial development of products/tests/treatments and I will not benefit financially from this. |  |
| 1. I agree to take part in the above study. |  |
| **The following items are optional. You may participate regardless of your response.** | |
| 1. I agree to being contacted in the future about participating in an interview about my experience of the JAKPPPOT trial. I understand that this part of the study is entirely optional. |  |
| 1. I agree to being contacted in the future if you need further clinical information and/or to inform me of future studies which may be of interest. I understand that these future studies are entirely optional. |  |

⎯⎯⎯⎯⎯⎯⎯⎯⎯⎯⎯⎯⎯⎯ ⎯⎯⎯⎯⎯⎯⎯⎯⎯⎯⎯ ⎯⎯⎯⎯⎯⎯⎯⎯⎯

Signature Name of Participant Date (DD/MMM/YYYY

⎯⎯⎯⎯⎯⎯⎯⎯⎯⎯⎯⎯⎯⎯ ⎯⎯⎯⎯⎯⎯⎯⎯⎯⎯⎯ ⎯⎯⎯⎯⎯⎯⎯⎯⎯

Signature Name of Researcher Date (DD/MMM/YYYY)

**Please keep original signed form in the Investigator Site File, and provide a copy for the medical notes and for the participant.**
